# Influence of the Coupling between Two Qubits in an Open Coherent Cavity: Nonclassical Information via Quasi-Probability Distributions

**DOI:** 10.3390/e21121137

**Published:** 2019-11-21

**Authors:** Abdel-Baset A. Mohamed, Hichem Eleuch, Abdel-Shafy F. Obada

**Affiliations:** 1 Department of Mathematics, College of Science and Humanities, Prince Sattam bin Abdulaziz University, Al-Aflaj 11942, Saudi Arabia; 2 Faculty of Science, Assiut University, Assiut 71515, Egypt; 3 Department of Applied Sciences and Mathematics, College of Arts and Sciences, Abu Dhabi University, Abu Dhabi 59911, UAE; hichem.eleuch@adu.ac.ae; 4 Faculty of Science, Al-Azhar University, Nasr City 11884, Egypt; asobada@yahoo.com

**Keywords:** intrinsic decoherence, quasi-probability distributions

## Abstract

In this paper, we investigate the dynamics of two coupled two-level systems (or qubits) that are resonantly interacting with a microwave cavity. We examine the effects of the intrinsic decoherence rate and the coupling between the two qubits on the non-classicality of different system partitions via quasi-probability functions. New definitions for the partial Q-function and its Wehrl entropy are used to investigate the information and the quantum coherence of the phase space. The amount of the quantum coherence and non-classicality can be appropriately tuned by suitably adopting the rates of the intrinsic-decoherence and the coupling between the two qubits. The intrinsic decoherence has a pronounced effect on the negativity and the positivity of the Wigner function. The coupling between the two qubits can control the negativity and positivity of the quasi-probability functions.

## 1. Introduction

Two-level system (qubit) is not only the key element in various fields of the modern physics, such as quantum optics and collision physics [1,2], but also the fundamental building block of modern applications ranging from quantum control [3] to quantum processing [4]. Such qubit systems were proposed for superconducting circuits [5,6], where both the 2-photon processes and two-qubit coupling have been realized [5,6,7]. Due to the rapid development of experiments in macroscopic solid-state physics, the artificial two-level atoms qubits, based on the superconducting (SC) circuits [8,9] and quantum dots (QDs) [10], have been recognized as possible candidate for the quantum processing.

Quasi-probability distributions (QPDs), Wigner function (WF) [11], and Q-function (QF) [12] are known to be valuable tools to explore the nonclassical features. The WF [13,14] may have negative part for some quantum states. The negative part of WF is a sufficient, but not necessary, condition for non-classicality of a quantum state; more precisely, a state with its WF being negative part is a non-classical state, whereas, for example, a squeezed state can be non-classical state with a non-negative WF. Therefore, the negative part of WF is used as an indicator for the non-classicality of a quantum state [15,16,17,18].

The Q-function is one of the simplest QPDs in phase space, and it is always a positive distribution [19]. Q-function and its Wehrl entropy can be used to quantify the quantum correlations and phase space information [20,21]. The Q-function was studied only for representations of the cavity fields, two-level systems [19,20,21], and three-level systems [22,23].

There are other information entropies, including von Neumann entropy [24], linear entropy, and Shannon information entropy [25], that are used as a measure for the entanglement in closed systems [10]. For open systems, they are used as a measure of the coherence loss, where there are two quantum coherence sources in a quantum system: The first is due to its unitary interaction, this coherence can be called entanglement between two subsystems and “coherence loss” for one of them. The second is in open systems, it leads to losing the quantum coherence. Wehrl entropy is an additional entropic quantity that has been successfully applied in measuring the entanglement. It gives quantitative and qualitative information on the entanglement of the bipartite system. It is a useful tool to investigate the dynamical properties that contain all the information about the system dynamics. It is proved that the Wehrl entropy exhibits a temporal evolution similar to the von Neumann entropy.

Loss of coherence in open quantum systems is as familiar as decoherence [26,27]. An important type of decoherence in open quantum systems is that described by the intrinsic decoherence (ID) model, in which Milburn [28] considers that the system does not evolve continuously, where the quantum coherence is automatically destroyed as the system evolves. The Milburn model is only used to investigate analytically decoherence effects on the dynamics of the non-classicality of the QPDs in two qubits when they are interacting with a coherent cavity field [29]; however, the effect of the coupling constant between the two qubits is neglected.

In the current work, (i) the physical model of two coupled two-level systems resonantly interacting with an open cavity initially in a superposition of coherent states is analytically described; (ii) we investigate the dynamic behaviors of the negativity and positivity of WF; and (iii) we introduce new definition for the partial Q-function of the two qubits and its Wehrl entropy.

The paper is organized as follows. In Section 2, the theoretical physical model and the time-dependent density matrices of the system are presented, and the definitions of the quasi-probability functions, the WF, and the two-qubit QF with its Wehrl entropy are considered in Section 3. The numerical analysis and related discussions on the quasi-probability functions are reported in Section 4. Finally, the conclusion is presented in Section 5.

## 2. Physical Model and Density Matrix

Here, we consider two coupled qubits that are resonantly interacting with a microwave cavity field via two atomic transitions. Such qubit systems were proposed in superconducting circuits [5,6], where the both 2-photon processes and two-qubit coupling have been realized [5,6,7]. In the rotating-wave approximation, the total Hamiltonian is

(1) $$\begin{array}{ccc} \hat{H} & = & \omega {\hat{a}}^{†}\hat{a}+\sum_{i=A,B}\left[\frac{\omega }{2}{\hat{\sigma }}_{i}^{z}+\lambda \left({\hat{a}}^{2}{\hat{\sigma }}_{i}^{+}+{\hat{a}}^{†2}{\hat{\sigma }}_{i}^{-}\right)\right]+J\left({\hat{\sigma }}_{A}^{+}⊗{\hat{\sigma }}_{B}^{-}+{\hat{\sigma }}_{A}^{-}⊗{\hat{\sigma }}_{B}^{+}\right),\; \end{array}$$

where ${\hat{a}}^{†}$ and $\hat{a}$ represent the raising and lowering operators of the microwave cavity field with the frequency ω, respectively. λ is the coupling constant strength between the cavity field and the qubits, whereas ${\hat{\sigma }}_{i}^{z}$, ${\hat{\sigma }}_{i}^{+}$, and ${\hat{\sigma }}_{i}^{-}$ represent the inversion, and the raising and the lowering operators of the *i*-th qubit, respectively, which are spanned by the $|1_{i}\rangle$ and $|0_{i}\rangle$(i=A,B), representing the excited and ground states. *J* is the coupling constant between the two qubits. There are two free parameters λ and *J* in the Hamiltonian. In our study, these free parameters do not lead to a kind of quantum phase transition [30,31,32,33] in the considered system. Where these parameters are not generating a sharp transition from a quantum phase to another quantum phase.

Here, we consider an important type of decoherence in open quantum systems is that described by the the intrinsic decoherence (ID) model. The time evolution of the ID model is governed by the master equation [28] as

(2) $$\begin{array}{c} \frac{d\hat{\rho }\left(t\right)}{dt}+\frac{i}{\hbar }\left[\hat{H},\hat{\rho }\left(t\right)\right]+\frac{\gamma }{2\hbar^{2}}\left[\hat{H},\left[\hat{H},\hat{\rho }\left(t\right)\right]\right]=0, \end{array}$$

where γ is the coupling constant of the intrinsic noise. For the sake of simplicity, we take here ℏ=1. The considered initial state of the system is

(3) $$\begin{array}{ccc} \hat{\rho }\left(0\right) & = & \sum_{m,n=0}^{\infty }\eta_{m}\eta_{n}^{*}|1_{A}1_{B},m\rangle \langle 1_{A}1_{B},n|,\;\eta_{m}=\frac{\left[1+{\left(-1\right)}^{m}\right]\mu^{m}e^{-\frac{1}{2}{\left|\mu \right|}^{2}}}{\sqrt{2m!(1+e^{-2\mu^{2}})}}. \end{array}$$

where the initial state of the cavity field is the even coherent state and μ is the amplitude of the coherent state. The two qubits are initially prepared in the state $|1_{A}1_{B}\rangle$.

In the space states $\{|1\rangle =|1_{A}1_{B},n\rangle ,|2\rangle =|1_{A}0_{B},n+2\rangle ,|3\rangle =|0_{A}1_{B},n+2\rangle ,|4\rangle =|0_{A}0_{B},n+4\rangle \;\}$, the dressed states $|\Psi_{i}^{n}\rangle$ and their eigenvalues of Equation (1) are

(4) $$\begin{array}{c} \left(\begin{array}{c} |\Psi_{1}^{n}\rangle \\ |\Psi_{2}^{n}\rangle \\ |\Psi_{3}^{n}\rangle \\ |\Psi_{4}^{n}\rangle \end{array}\right)=P\left(\begin{array}{c} |1\rangle \\ |2\rangle \\ |3\rangle \\ |4\rangle \end{array}\right),\;\;P=\left(\begin{array}{cccc} \bar{y} & 0 & 0 & -\bar{x}\\ 0 & 1/\sqrt{2} & -1/\sqrt{2} & 0\\ 2{\bar{x}}_{-} & -0.5{\bar{D}}_{-} & -0.5{\bar{D}}_{-} & 2{\bar{y}}_{-}\\ 2{\bar{x}}_{+} & 0.5{\bar{D}}_{+} & 0.5{\bar{D}}_{+} & 2{\bar{y}}_{+} \end{array}\right), \end{array}$$

(5) $$\begin{array}{ccc} E_{1}^{n} & = & \omega \left(n+1\right)\;E_{2}^{n}=\omega \left(n+1\right)-J,\\ E_{3(4)}^{n} & = & \omega \left(n+1\right)+\frac{1}{2}J\mp R,\;R=\sqrt{J^{2}+8\left(x^{2}+y^{2}\right)}. \end{array}$$

with

$$\begin{array}{ccc} \;\;\;\;\;\bar{x} & = & \frac{x}{\sqrt{x^{2}+y^{2}}},\bar{y}=\frac{y}{\sqrt{x^{2}+y^{2}}},{\bar{x}}_{\pm }=\frac{x}{\chi_{\pm }},{\bar{y}}_{\pm }=\frac{y}{\chi_{\pm }},\chi_{\pm }=\sqrt{RD_{\pm }},\\ \;\;\;\;\;{\bar{D}}_{\pm } & = & D_{\pm }/\chi_{\pm },D_{\pm }=R\pm J,x=\lambda \sqrt{\left(n+1)(n+2\right)},y=\lambda \sqrt{\left(n+3)(n+4\right)}. \end{array}$$

Using Equation (2), the time evolution for the dressed state matrices, $|\Psi_{i}^{m}\rangle \langle \Psi_{j}^{n}|$, are given by

(6) $$\begin{array}{ccc} |\Psi_{i}^{m}\rangle \langle \Psi_{j}^{n}{|}_{t}\;\; & = & e^{-i\lambda \left(E_{i}^{m}-E_{j}^{n}\right)t-\gamma {\left(E_{i}^{m}-E_{j}^{n}\right)}^{2}t}|\Psi_{i}^{m}\rangle \langle \Psi_{j}^{n}{|}_{t=0}, \end{array}$$

where $E_{i}^{m}$ are the eigenvalues of the dressed states $|\Psi_{i}^{m}\rangle$. From the initial density matrix and using Equation (5), the final expression of the density matrix is then

(7) $$\begin{array}{cc} \hat{\rho }\left(t\right) & =\sum_{m,n=0}\eta_{m}\eta_{n}\;\;\{\alpha_{11}|\Psi_{1}^{m}\rangle \langle \Psi_{1}^{n}{|}_{t}+\alpha_{31}|\Psi_{3}^{m}\rangle \langle \Psi_{1}^{n}{|}_{t}+\alpha_{41}|\Psi_{4}^{m}\rangle \langle \Psi_{1}^{n}{|}_{t}\\ & +\alpha_{13}|\Psi_{1}^{m}\rangle \langle \Psi_{3}^{n}{|}_{t}+\alpha_{33}|\Psi_{3}^{m}\rangle \langle \Psi_{3}^{n}{|}_{t}+\alpha_{43}|\Psi_{4}^{m}\rangle \langle \Psi_{3}^{n}{|}_{t}+\alpha_{14}|\Psi_{1}^{m}\rangle \langle \Psi_{4}^{n}{|}_{t}\\ & +\alpha_{34}|\Psi_{3}^{m}\rangle \langle \Psi_{4}^{n}{|}_{t}+\alpha_{44}|\Psi_{4}^{m}\rangle \langle \Psi_{4}^{n}{|}_{t}\;\;\}, \end{array}$$

where $\alpha_{mn}=P_{m1}P_{n1}$, $P_{ij}$ are the elements of the matrix $P=[P_{ij}]$ of Equation (4).

In this work, we focus on the non-classicality feature of the different system partitions, the quasi-probability distribution of WF and QF. We consider the following number states, |1〉=|n〉,|2〉=|3〉=|n+2〉,|4〉=|n+4〉. The matrix representation of the cavity field, $\rho^{f}\left(t\right)$, is

(8) $$\begin{array}{ccc} \;\;\;\;\rho^{f}\left(t\right) & = & {\mathrm{Tr}}_{\mathrm{qubits}}\left\{\rho \left(t\right)\right\}=\sum_{m,n=0}^{\infty }\sum_{k=1}^{4}\rho_{kk}^{mn}|k\rangle \langle k|, \end{array}$$

where $\rho_{kk}^{mn}$ are the elements of $\rho^{f}\left(t\right)$. The matrix representation of the two-qubit system is

(9) $$\begin{array}{c} {\hat{\rho }}^{AB}\left(t\right)={\mathrm{Tr}}_{\mathrm{field}}\left\{\rho \left(t\right)\right\}=\sum_{n=0}^{\infty }\langle n|\hat{\rho }\left(t\right)|n\rangle , \end{array}$$

which is used for computing the matrix representations of other partitions of the *i*-qubit (i=A,B) by $\rho^{A(B)}\left(t\right)={\mathrm{Tr}}_{B(A)}\left\{\rho^{AB}\left(t\right)\right\}$.

## 3. Quasi-Probability Distributions

A useful criterion to determine the non-classical features for the quantum states is to represent these states in phase space according to the QPDs. The *s*-QPDs for a state $\hat{\rho }\left(t\right)$ are given by [34,35]

(10) $$F\left(\alpha ,s\right)=\frac{2}{\pi }\sum_{n=0}^{\infty }{\left(-1\right)}^{n}\frac{{\left(1+s\right)}^{n}}{{\left(1-s\right)}^{n+1}}\langle \alpha ,n|\hat{\rho }\left(t\right)|\alpha ,n\rangle .$$

If s=0,−1, then F(α,s) represents WF and QF, respectively. The state |α,n〉=D(α)|n〉 designates the displaced number state with the displacement operator $D\left(\alpha \right)=e^{\left(\alpha {\hat{a}}^{+}-\alpha^{*}\hat{a}\right)}$. The amplitude of the displaced number state in the Fock number representation is given by [36]

(11) $$f_{m,n}=\langle m|\alpha ,n\rangle =\sqrt{\frac{n!}{m!}}\alpha^{m-n}e^{-\frac{1}{2}{\left|\alpha \right|}^{2}}L_{n}^{m-n}{\left(|\alpha \right|}^{2},$$

where $L_{n}^{m-n}{\left(|\alpha \right|}^{2})$ is the associated Laguerre polynomial. As the *s*-parameterized QPDs depend on the elements of the density matrix of the subsystems, these implicitly give the phase space information about all their operators.

### 3.1. Wigner Function

The investigation of the negative and positive parts of WF is an important tool to quantify the non-classicality of a quantum system, where some non-classical information may be expected intuitively via its negative part. Therefore, we can determine the non-classicality under the influence of both the ID and the coupling between the two qubits via the negative part of the WF. In the field coherent state representation |α〉, the WF of the cavity field of Equation (8) can be expressed as [34,35]

(12) $$\begin{array}{cc} W(\alpha )= & \frac{2}{\pi }\sum_{l=0}^{\infty }\sum_{m,n=0}^{\infty }[\rho_{11}^{mn}f_{l,m}^{*}f_{n,l}+\rho_{44}^{mn}f_{l,m+2}^{*}f_{n+2,l}\\ & +\left(\rho_{22}^{mn}+\rho_{33}^{m,n}\right)f_{l,m+1}^{*}f_{n+1,l}]{\left(-1\right)}^{l}. \end{array}$$

### 3.2. Q-Function and Partial Wehrl Entropies

For the total angular momentum number $j=\frac{1}{2}$, the Bloch coherent quantum state of *k*-qubit (k=A,B)$|\theta_{k},\phi_{k}\rangle$ [37] is

(13) $$|\theta_{k},\phi_{k}\rangle =\cos\frac{\theta_{k}}{2}\;|1_{k}\rangle +e^{i\phi_{k}}\sin\frac{\theta_{k}}{2}\;|0_{k}\rangle .$$

The phase space can be determined by its angles, (θ,ϕ), with θ∈[0,π] and ϕ∈[0,2π]. Therefore, the Q-function of the *k*-qubit is given by [12]

(14) $$\begin{array}{ccc} Q^{k}\left(\theta_{k},\phi_{k},t\right) & = & \frac{1}{2\pi }\langle \theta_{k},\phi_{k}|\rho^{k}\left(t\right)|\theta_{k},\phi_{k}\rangle . \end{array}$$

In the two-qubit phase space represented by $\{|1_{A}1_{B}\rangle ,|1_{A}0_{B}\rangle ,|0_{A}1_{B}\rangle ,|0_{A}0_{B}\rangle \}$, the Bloch coherent of the two-qubit states is defined as

(15) $$\begin{array}{ll} {|\Phi \rangle }_{AB}= & \cos\frac{\theta_{A}}{2}\cos\frac{\theta_{B}}{2}|1_{A}1_{B}\rangle +e^{i\phi_{B}}\cos\frac{\theta_{A}}{2}\sin\frac{\theta_{B}}{2}|1_{A}0_{B}\rangle \\ & \;+e^{i\phi_{A}}\sin\frac{\theta_{A}}{2}\cos\frac{\theta_{B}}{2}|0_{A}1_{B}\rangle +e^{i(\phi_{A}+\phi_{B})}\sin\frac{\theta_{A}}{2}\sin\frac{\theta_{B}}{2}|0_{A}0_{B}\rangle . \end{array}$$

Therefore, the QF of the two-qubit system $\rho^{AB}\left(t\right)$ is given by

(16) $$\begin{array}{ccc} Q^{AB}\left(\Phi ,t\right) & = & \frac{1}{4\pi^{2}}\langle \Phi |\rho^{AB}\left(t\right){|\Phi \rangle }_{AB}, \end{array}$$

and its partial QFs of the *k*-qubit (say k=A) are given by

(17) $$\begin{array}{ccc} Q^{A}\left(\theta_{A},\phi_{A},t\right) & = & \int_{0}^{\pi }\int_{0}^{2\pi }Q^{AB}\left(\Phi ,t\right)\sin\theta_{B}\;d\phi_{B}\;d\theta_{B}. \end{array}$$

Wehrl entropy is one of Q-function applications [38,39]. Here, it is used to measure the coherence loss in open systems and entanglement in closed systems, which are useful in quantum information and computation [40]. The partial Wehrl entropies of the *k*-qubit are given by

(18) $$\begin{array}{ccc} \;\;\;\;E^{k}\left(t\right) & = & \int_{0}^{2\pi }\int_{0}^{\pi }D^{k}\left(\theta ,\phi ,t\right)\;\sin\theta \;d\theta \;d\phi . \end{array}$$

where the partial Wehrl density of the *k*-qubit, $D^{k}\left(\theta ,\phi ,t\right)$, is given by

(19) $$\begin{array}{c} D^{k}\left(\theta ,\phi ,t\right)=-Q^{k}\left(\theta ,\phi ,t\right)\;\ln\left[Q^{k}\left(\theta ,\phi ,t\right)\right]. \end{array}$$

Here, the phase space information is the QF distribution of the two-qubit system in the space formed by angles θ and ϕ [22,23]. Wehrl density is used as a useful measure for the loss of information about the *k*-qubit state due to ID, where the loss of information is independent of the phase space parameters. Note that the Wehrl entropy cannot be negative. As it is generally difficult to find a closed form for the Wehrl entropy, numerical techniques must be used. If the two qubits are initially prepared in the state $|1_{A}1_{B}\rangle$,

(20) $$\begin{array}{ccc} \;\;\;\;E^{A}\left(0\right) & = & -2\int_{0}^{\pi /2}\sin2\theta \cos^{2}\theta \ln\left[\cos^{2}\left(\theta \right)/2\pi \right]\;d\theta =2.3379, \end{array}$$

it grows in time as [22,39]

$$2.3379\leq E^{A}\left(t\right)\leq \ln\left(4\pi \right).$$

## 4. Numerical Analysis of Quasi-Probability Functions

Here, we explore the non-classicality of the WF and QF with particular choices of the parameters of γ/λ of ID rate; the coupling constants of both unitary interactions, λt; and J/λ of the interaction between the two qubits. To explore the unitary interaction effect, we study both cases λt=0 (the initial non-classicality) and λt>0 (the dynamical of the non-classicality). To explore the effect of the intrinsic noise and the interaction between the two qubits, we vary the parameters γ/λ and J/λ. The positivity and negativity of WF are analyzed through the phase space parameter α=x+iy. There are particular values of the parameters of the phase space α=x+iy that allow to analysis the positivity and negativity of WF.

### 4.1. Numerical Analysis of WF

**Figure 1:**
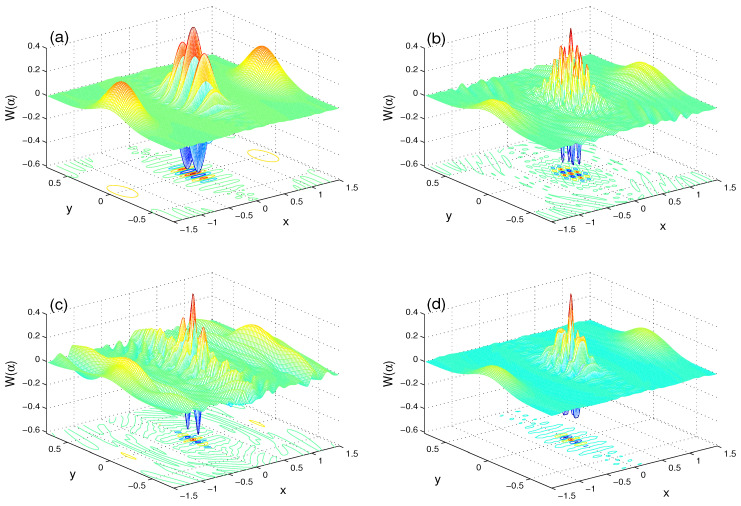
Wigner function with μ=4 for λt=0.0 (**a**); (λt,J,γ)=(2.25π,0,0) (**b**); (λt,J,γ)=(2.2π,30λ,0) (**c**); and (λt,J,γ)=(2.2π,0,0.1λ) (**d**).

**Figure 2:**
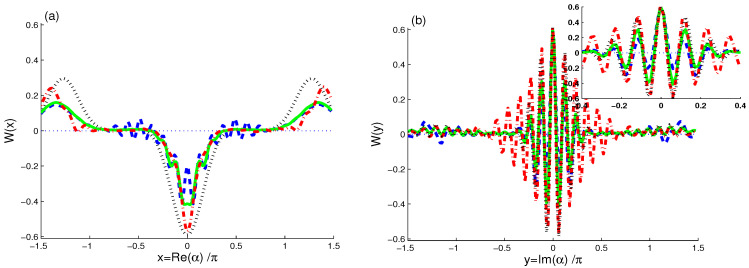
W(α=x+iy) at (**a**) Im(α)=−0.06127π and (**b**) Re=0.009296π for different cases (λt,J,γ)=(0,0,0) (dot curves), (λt,J,γ)=(2.2π,0,0) (dash curves), (λt,J,γ)=(2.2π,30λ,0) (dash-dot curves), and (λt,J,γ)=(2.2π,0,0.1λ) (sold curves).

**Figure 3:**
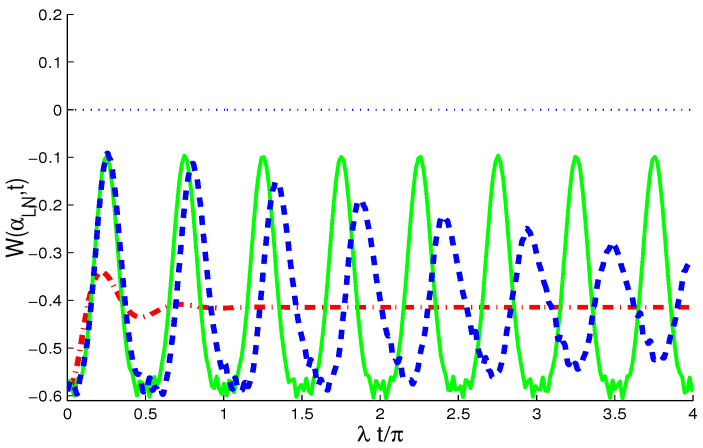
The $W(\alpha_{LN},t)$ with different cases (J,γ)=(0,0) (sold curves), (J,γ)=(30λ,0) (dash curves), and (J,γ)=(0,0.1λ) (dash-dot curves).

We depict the WF, W(α,t), in the complex plane α=x+iy: x∈[−1.5π,1.5π] and y∈[−0.75π,0.75π]. In Figure 1, Figure 2 and Figure 3, we present the behavior of the W(α) and its marginal functions.

In Figure 1, the W(α) is meshed for different cases, (λt,J,γ): (λt,J,γ)=(0,0,0) in panel (a), (λt,J,γ)=(2.25π,0,0) in panel (b), (λt,J,γ)=(2.25π,30λ,0) in panel (c), and (λt,J,γ)=(2.25π,0,0.1λ) in panel (d). In Figure 1a, the WF is negative over large regions in the phase space. It has two primary minima (their depth reaches approximately −0.6) and secondary minima under the zero-level. This means that the negativity part is a natural signature of the non-classicality. Also, the WF has some maxima, which confirm the positivity of its distribution.

The effect of the unitary interaction on the initial maxima and minima of WF is considered via the parameter λt: λt=0 in Figure 1a and λt=2.25π in Figure 1b. For this value of λt, the negativity and positivity of the WF distribution are reduced.

In Figure 1c, the dependence of the heights and depths of the generated interference has maxima and minima on the rate J/λ of the coupling between the two qubits, as shown in the case (λt,J,γ)=(2.25π,30λ,0). We note that reduced maxima and minima, due to the unitary interaction, are enhanced.

The dependence of the negativity and positivity features of WF on the decoherence rate γ/λ is presented in Figure 1c with the case (λt,J,γ)=(2.2π,0,0.1λ). The positive and negative parts of W(α) are reduced. From Figure 1b,c, we deduce that this rate enhances the stationary value for the positive and negative parts. Moreover, with ID, these parts do not vanish completely.

In Figure 2, the functions W(x,−0.06127) and W(0.009296,y) against the x=Re(α)/π and y=Im(α)/π of the lowest negative value of W(α), (0.009296π,−0.06127π), respectively, occur for different cases: (λt,J,γ)=(0,0,0), (λt,J,γ)=(2.2π,0,0), (λt,J,γ)=(2.2π,30λ,0), and (λt,J,γ)=(2.2π,0,0.1λ). Note that the largest negative (LN) value of $W(\alpha_{LN},t)$ is at $\left(\alpha_{LN}=0.009296\pi -0.06127\pi i\right)$.

The dot-curve in Figure 2a shows that positive part of W(x) always appears as two symmetrical well-separated unconstrained Gaussian maxima, whereas the negative part appears as one remarkable minima. These maxima and minima have symmetry around x=0. When the interaction between the cavity and the qubits is started, at value λt=2.2π, the ID rate is considered as 0.1λ and the upper and lower points of the maxima and minima of WF reduce, see the dash and sold curves. The dash-dot curve in Figure 1c shows the dependence of the heights and depths of these maxima and minima on the coupling rate J/λ between the two qubits. The non-classicality can be enhanced by increasing the J/λ.

In Figure 2b, the dot-curve of W(y) is plotted at fixed value of x=0.009296π for the same case, (λt,J,γ)=(0,0,0). The dot curve shows that the negativity and positivity of WF have regular fluctuations as revivals and collapses in the phase space with more oscillations. For the non-zero values of the unitary interaction parameter λt and ID rate γ/λ, the oscillations and their amplitudes become weaken compared to (λt,J,γ)=(0,0,0). The maxima and minima and their heights and depths depend on the values λt and γλ, whereas the increase of the coupling between two qubits, J/λ, enhances the oscillations and their amplitudes. The non-classicality could be improved by increasing the rate J/λ.

Figure 3 shows the time evolution for the largest negative value of $W(\alpha_{LN},t)$, corresponding to $\left(\alpha_{LN}=0.009296\pi -0.06127\pi i\right)$, for different cases (J,γ)=(0,0) (sold curve), (J,γ)=(30λ,0) (dash curve), and (J,γ)=(0,0.1λ) (dash-dot curve) when μ=4. In the case of (J,γ)=(0,0), the temporal evolution of $W(\alpha_{LN},t)$ shows regular fluctuations between its extrema. The WF function oscillates with a π/2-period, and it conserves the non-classicality. We note that the maxima of the non-classicality are at $\frac{(2n+1)\pi }{4},\left(n=0,1,2,...\right)$, whereas the minima are at $\frac{n\pi }{2},\left(n=0,1,2,...\right)$.

From the dash curve for the case (J,γ)=(30λ,0), we observe that the coupling between two qubits affects only on the maxima and minima of $W(\alpha_{LN},t)$, and the difference between them decreases.

From the case of (J,γ)=(0,0.1λ) (dash-dot curves), the ID rate leads to notable changes in the extreme values of the WF. This confirms that, with the ID rate γ/λ, the oscillations are dampened and $W(\alpha_{LN},t)$ reaches its stationary value ($W(\alpha_{LN},\infty )=-0.4143$) more rapidly. After a short time, λt>0.8π, the ID destroys the oscillations completely and WF reaches its stationary value. The temporal evolution of the negative values is hypersensitive to the rates of the ID and the coupling between the two qubits.

### 4.2. Numerical Analysis of QF

**Figure 4:**
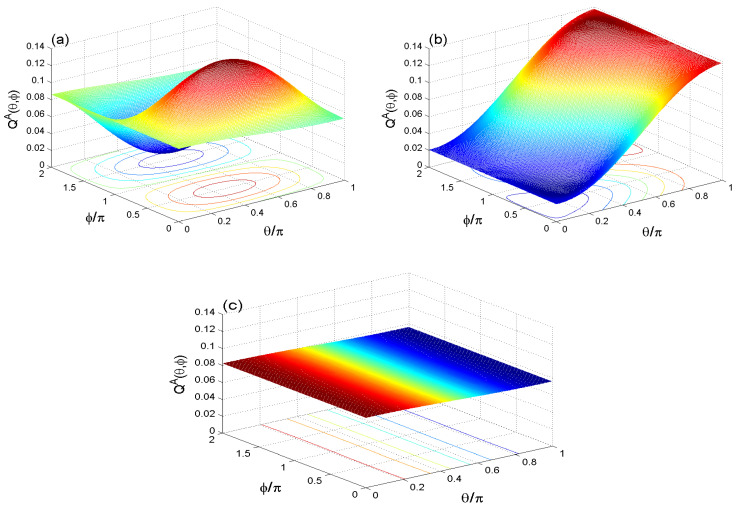
$Q^{A}\left(\theta ,\phi ,t\right)$ at λt=2.011π and μ=4 with different cases of (J,γ)=(0,0) (**a**); (J,γ)=(10λ,0) (**b**); and (J,γ)=(0,0.05λ) (**c**).

At λt=2.011π, the partial Q-function $Q^{A}\left(\theta ,\phi \right)$ is plotted in the phase space θ∈[0,π] and ϕ∈[0,2π] for μ=4 in Figure 4 with different cases (J,γ): (J,γ)=(0,0) in panel (a), (J,γ)=(10λ,0) in panel (b), and (J,γ)=(0,0.01λ) in panel (c). We note that the $Q^{A}\left(\theta ,\phi \right)$ is distributed regularity on the phase space (θ,ϕ).

From Figure 4b, the dependence of $Q^{A}\left(\theta ,\phi \right)$ on the rate of coupling between two qubits, J/λ, is shown. We observe that standard-level value of $Q^{A}\left(\theta ,\phi \right)$ of the case of J=10λ drops from ≃0.086-level to ≃0.02-level due to the increasing of the coupling rate between the two qubits. The information of QF may be controlled with the coupling rate of the two qubits.

In Figure 4c, the effect of the ID rate on the $Q^{A}\left(\theta ,\phi \right)$, γ=0.05λ, is shown. We observe that the dissipation rate causes notable changes in the heights and depths of the generated peaks and troughs of QF.

In Figure 4, the counter of $Q^{A}\left(\theta ,\phi \right)$ appears as color curves in the θ−ϕ plane. The increase of ID rate from γ=0.0 to γ=0.05λ, converts the elliptic curves to lines, i.e., the Q-function appears as a constant value in function of the two angles due to the ID.

### 4.3. Coherence Loss of Wehrl Entropy

**Figure 5:**
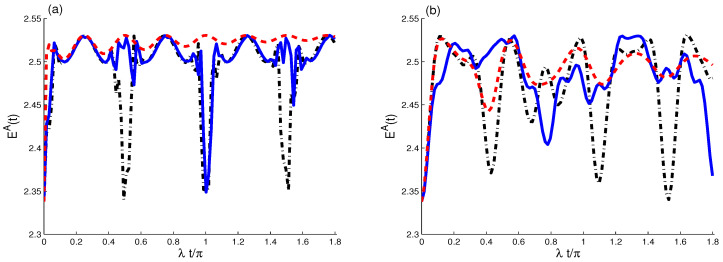
Wehrl entropy $E^{A}\left(t\right)$ for different cases μ: μ=4 (**a**) and μ=1 (**b**), with different cases (J,γ)=(0,0) (dot curves), (J,γ)=(10λ,0) (sold curves), and (J,γ)=(0,0.01λ) (dash curves).

The partial Wehrl entropy, $E^{A}\left(t\right)$, of *A*-qubit is used to quantify their phase space coherence loss. In Figure 5a, the function $E^{A}\left(t\right)$ is plotted with different cases: (J,γ)=(0,0) (dot curves), (J,γ)=(10λ,0) (sold curve), and (J,γ)=(0,0.01λ) (dash curves) for large value μ=4. $E^{A}\left(t\right)$ has oscillatory behavior; it oscillates periodically with a $\frac{\pi }{2}$-period. This means that the quantum coherence is lost and gained regularly. The effect of the coupling rate, J/λ, on the behavior of $E^{A}\left(t\right)$ is shown in the sold curve. With J=10λ, the partial Wehrl entropy have large period and its minimum values decrease.

The effect of the ID rate is plotted in the dash curve with (J,γ)=(0,0.01λ). The Wehrl entropy has damped oscillatory behavior and reaches stationary value after short time, i.e., the *A*-qubit reaches mixture state and it completely loses its quantum coherence after a defined time. In Figure 5b, the case of small value, μ=1, we note that the amplitude of the coherent state leads to more oscillations and coherence loss. It reduces the effect of the ID rate.

## 5. Conclusions

In the current work, we analyzed two coupled qubits resonantly interacting with a cavity field via two atomic transitions. The positive and negative parts of the WF and the Q-function, with its partial Wehrl entropies, are used to investigate the non-classicality. The rates of ID and the coupling between the two qubits have pronounced effects on the negativity and positivity of the Wigner function. The results are shown that the partial distributions of QF, and their phase space information depends on the rates of the ID and the coupling between the two qubits. The coherence loss of the states of of each qubit, due to the cavity–qubit interaction, is studied via its partial Wehrl entropy. The results show that the partial Wehrl entropies and their steady-state values depend on the amplitude of the coherent state and the ID rate, as well as the coupling between the two qubits.

The control of the non-classicality and quantum coherence via the physical parameters opens the door to the conception of optical states with unconventional proprieties. It has been widely recognized that quantum effects (including non-classicality and quantum coherence) are valuable resources for quantum information [10].
